# Speed breeding in upland cotton (*Gossypium hirsutum* L): scientific foundations and integrated implementation of a next-generation cotton breeding platform

**DOI:** 10.3389/fpls.2026.1915693

**Published:** 2026-09-07

**Authors:** Abrorjon Y. Kurbonov, Feruza F. Mamedova, Rayhona M. Isayeva, Ural A. Khamirayev, Shengli Liu, Wang Lizhi, Sabir P. Makhmadjanov, Amandyk K. Kostakov, Amangeldy I. Aliyev, Olzhas A. Kostak, Djanibek S. Makhmadjanov

**Affiliations:** 1 Cotton Biotechnology Laboratory, Research Institute of Cotton Breeding, Seed Production and Agrotechnologies (CBSPARI), Tashkent, Uzbekistan; 2 Fruit Crop Biotechnology Laboratory, Academician M. Mirzaev Research Institute of Horticulture, Viticulture and Winemaking, Tashkent, Uzbekistan; 3 State Key Laboratory of Cotton Bio-Breeding and Integrated Utilization, School of Agriculture and Biomanufacturing, Zhengzhou University, Zhengzhou, China; 4 State Key Laboratory of Cotton Biology, Institute of Cotton Research of the Chinese Academy of Agricultural Sciences, Anyang, China; 5 Agricultural Experimental Station of Cotton and Melon Growing, Atakent, Kazakhstan

**Keywords:** CRISPR, GASB, genomic selection, *Gossypium hirsutum*, immature embryo rescue, integrated breeding system, LED photobiology, salt tolerance

## Abstract

Conventional cotton (*Gossypium* spp.) variety development requires 8–12 years - a timeline structurally incompatible with the accelerating urgency of climate-change adaptation, soil salinisation, and textile-industry demands for novel varieties within 3–5 years. Speed breeding (SB) compresses the generation interval by 30–80% through synergistic optimisation of LED spectra, temperature, nutrition, and immature embryo rescue (IER). However, SB alone is a temporal accelerator, not a breeding system: its transformative potential is realised only when it functions as the central engine of a genomics-assisted speed breeding (GASB) platform that integrates molecular marker-assisted selection (MAS), genomic selection (GS), CRISPR and New Genomic Technique (NGT) editing, high-throughput phenotyping (HTP), and AI-assisted decision-support tools. Building on previous reviews of cotton speed breeding and genomics-assisted breeding, the present review provides a mechanistic and implementation-oriented synthesis of these complementary technologies within a unified GASB framework. We evaluate: (i) the molecular architecture of photoperiodic flowering in predominantly *Gossypium hirsutum*, together with currently available evidence from other *Gossypium* species where appropriate and its manipulation through LED protocol design and CRISPR targeting of flowering suppressors; (ii) the mechanistic basis of multi-channel LED recipe design from SB 1.0 to SB 3.0; (iii) stage-specific thermal management including the operationally critical 33°C pollen safety ceiling; (iv) nutritional physiology, phytohormones, and biochemical monitoring; (v) *in vitro* biotechnologies—including callus culture, microspore-derived doubled haploids, and immature embryo rescue (IER)—together with the operational coordination of immature embryo rescue and pollen-stage temperature management within speed-breeding facilities; (vi) high-throughput phenotyping technologies experimentally validated in cotton, encompassing five cotton-specific applications reviewed in a dedicated section; and (vii) AI-assisted phenotyping and digital twin architectures for real-time decision support within the GASB pipeline. Critical analysis identifies allotetraploid genome redundancy, genotype × light environment (G×L) interaction with landrace germplasm, IER scalability, bioinformatics infrastructure gaps, SB-to-field correlation uncertainties, and SVT regulatory requirements as the primary barriers to widespread GASB deployment. A structured 2025–2035 roadmap with three time-horizon priorities and measurable KPIs is presented.

## Introduction

Cotton (*Gossypium* spp.) is one of the world’s most economically significant non-food crops. Global lint production reaches approximately 25 million tonnes per year, supporting a market valued at USD 43 billion and providing livelihoods for more than 175 million people across 75 producing nations (USDA-FAS, 2024; Renob Research, 2024). The crop faces a convergence of intensifying stresses that demand new, adapted varieties at unprecedented speed. Heat stress at combined day/night temperatures of 37/26°C reduces lint yield by 50–60% (Mishra et al., 2017; Saini et al., 2023; Bista et al., 2025; Akhunov et al., 2025). Soil salinisation is advancing across every major cotton-producing region: in Uzbekistan, more than 50% of irrigated agricultural land is salt-affected, and 68% of growing sites exceed the ECe >7 dS m-1 threshold at which significant yield penalties arise (Sultonov et al., 2025; Akhmedov et al., 2025). These challenges increase the demand for new varieties within 3–5 years, whereas conventional breeding pipelines typically require 8–12 years. Soil-borne pathogens including *Verticillium* and *Fusarium* wilt impose further constraints, necessitating simultaneous improvement of resistance traits alongside climate adaptation (Kurbonov et al., 2026a, Kurbonov et al., 2026b).

The structural bottleneck in cotton improvement is generation interval (L). The breeder’s equation, **ΔG = (i × r × σA)/L**, states that annual genetic gain is inversely proportional to generation interval. Using a conventional field-based breeding system with one generation per year (approximately 365 days) as an illustrative reference, reducing the generation interval (L) to 102 days (LED-optimised speed breeding without IER) theoretically increases the potential rate of annual genetic gain by approximately 3.6-fold, assuming that selection intensity (i), selection accuracy (r), and additive genetic variance (σA) remain constant. Likewise, the five-generation-per-year cycle demonstrated in cotton (Wang et al., 2025) theoretically increases the potential rate of annual genetic gain by approximately 5.2-fold under the same assumptions. However, realised genetic gain also depends on factors such as selection accuracy, effective population size, maintenance of genetic diversity, genotype × environment interactions, and breeding strategy. Speed breeding attacks the denominator directly. Genomics-assisted speed breeding (GASB) can improve selection intensity (i) through molecular pre-screening and selection accuracy (r) through genomic prediction while facilitating more efficient utilisation of existing genetic diversity. However, the maintenance of additive genetic variance (σA) depends on breeding population management, mating design, and long-term selection strategy rather than genomic selection itself (Hickey et al., 2019; Voss-Fels et al., 2019; Raza et al., 2024).

Nevertheless, the baseline generation interval differs among breeding programmes because many cotton improvement programmes already use shuttle breeding, greenhouse generation advancement, or off-season nurseries to accelerate generation turnover. Therefore, the 365-day interval is presented only as an illustrative conventional field-based reference rather than a universal baseline.

The field has advanced rapidly. Watson et al. (2018) formalised SB protocols achieving up to six generations per year in wheat and barley. Ghosh et al. (2018) extended these to detailed multi-species protocols. For cotton specifically, Wang et al. (2025) achieved five generations per year in *G. hirsutum* using optimised LED spectra combined with IER, reducing the average breeding cycle from 130 days to 71–85 days. Rapid parallel advances in genomic selection for cotton fibre quality (Li et al., 2022; Khalilisamani et al., 2024), CRISPR and base-editing of fibre and architectural traits (Wang et al., 2024; Sheri et al., 2025; Qin et al., 2019), high-throughput phenotyping using deep learning (Rolland et al., 2022; Renó et al., 2024), and AI-driven digital twin breeding models (Crossa et al., 2021) collectively constitute a genuine paradigm convergence. Several recent reviews have summarised different aspects of cotton speed breeding and genomics-assisted breeding. However, the present review extends these studies by providing a mechanistic and implementation-oriented synthesis that integrates controlled-environment optimisation, LED photobiology, stage-specific temperature management, immature embryo rescue, molecular breeding technologies, genomic prediction, AI-assisted phenotyping, standardised validation strategies, and practical implementation pathways within a unified GASB framework.

Existing reviews have substantially advanced the field of cotton speed breeding and genomics-assisted breeding from complementary perspectives. Conaty et al. (2022) presented an integrated breeding framework combining genomic selection, gene editing, phenomics, pan-omics, artificial intelligence, and advanced breeding technologies. Baghyalakshmi (2023) highlighted the role of speed breeding under controlled environments together with immature embryo rescue, marker-assisted selection, genomic selection, gene editing, and phenomics. Khan et al. (2025) reviewed genomic approaches for climate-resilient cotton improvement, whereas Kutum et al. (2025) summarised recent advances in integrating conventional and molecular breeding technologies. Wang et al. (2025) further outlined future breeding strategies linking cotton genomics with optimised breeding systems.

Despite these important advances, several cotton-specific challenges remain insufficiently integrated, including allotetraploid genome redundancy requiring multiplex NGT strategies (Wang et al., 2017; Sheri et al., 2025), residual short-day sensitivity in non-elite germplasm (Abdurakhmonov et al., 2022; Zhu et al., 2024), coordination of immature embryo rescue with the tetrad-stage thermal vulnerability window, genotype-specific controlled-environment optimisation, and practical implementation of genomics-assisted speed breeding under routine breeding conditions.

Rather than replacing previous reviews, the present review complements and extends them by providing a mechanistic and implementation-oriented synthesis that integrates biological, physiological, molecular, and operational components within a unified GASB framework, with particular emphasis on LED photobiology, stage-specific temperature management, and practical implementation. To improve conceptual clarity, the evolution of speed breeding is summarised into three sequential development stages (**Table 1**). These stages do not represent rigid classifications but rather illustrate the progressive integration of complementary breeding technologies.

**Table 1:** Evolution of speed breeding concepts from SB 1.0 to SB 3.0 (genomics-assisted speed breeding, GASB).

Feature	SB 1.0	SB 2.0	SB 3.0 (GASB)
Primary objective	Faster generation advancement	Optimised speed breeding	Integrated genomics-assisted breeding
Main technologies	Extended photoperiod, controlled environment	Optimised lighting, SSD, improved protocols	SB + MAS + GS + AI + HTP + Digital twins
Selection strategy	Phenotypic selection	Phenotypic + limited markers	Genomic prediction + AI-assisted selection
Typical breeding stage	Generation advancement	Early generation selection	Whole breeding pipeline
Data integration	Minimal	Moderate	Multi-omics, phenomics, genomics and environmental data
Expected outcome	More generations per year	Faster and more reliable SB	SB 3.0 (GASB)

## Positioning of the present review within existing cotton breeding literature

During the past several years, several important reviews have summarised different aspects of modern cotton breeding. Conaty et al. (2022) outlined integrated breeding strategies involving genomic selection, gene editing, phenomics, pan-omics, artificial intelligence, and advanced breeding technologies. Baghyalakshmi (2023) discussed speed breeding together with controlled-environment cultivation, immature embryo rescue, marker-assisted selection, genomic selection, gene editing, and phenomics. Khan et al. (2025) reviewed genomic approaches for climate-resilient cotton improvement, whereas Kutum et al. (2025) summarised recent advances in molecular breeding technologies. Wang et al. (2025) presented future perspectives linking cotton genomics with optimised breeding strategies.

Rather than replacing these important reviews, the present review complements and extends them by emphasising the mechanistic integration of LED photobiology, stage-specific temperature management, immature embryo rescue, genomics-assisted speed breeding, standardised multi-environment validation, resource-based implementation pathways, AI-assisted phenotyping, digital twin concepts, and a quantitative roadmap for practical cotton breeding programmes.

As summarised in **Table 2**, previous reviews have made important contributions to individual aspects of cotton speed breeding and genomics-assisted breeding. Rather than replacing these valuable studies, the present review complements and extends them by integrating the biological, physiological, molecular, and operational components into a unified mechanistic and implementation-oriented GASB framework, with particular emphasis on LED photobiology, stage-specific temperature management, immature embryo rescue, standardised multi-environment validation, high-throughput phenotyping with five cotton-validated applications, AI-assisted digital twin architectures, and practical implementation pathways.

**Table 2:** Comparison of previous cotton speed breeding and genomics-assisted breeding reviews with the additional contributions of the present review.

Scope/feature	Conaty et al. (2022)	Baghyalakshmi (2023)	Khan et al. (2025)	Kutum et al. (2025)	Wang et al. (2025)	Present review
Cotton speed breeding	Brief overview	Primary focus	Brief	Moderate	Moderate	Comprehensive
Marker-assisted selection (MAS)	✓	✓	✓	✓	✓	✓
Genomic selection (GS)	✓	✓	✓	✓	✓	✓
Gene editing (CRISPR/NGTs)	✓	✓	✓	✓	✓	✓
High-throughput phenotyping	✓	Brief	✓	Brief	✓	Comprehensive
Artificial intelligence/machine learning	✓	–	✓	Brief	✓	Comprehensive
Digital breeding concepts	Brief	–	Brief	–	✓	✓
LED photobiology (mechanistic analysis)	–	Limited	–	–	–	Comprehensive
Stage-specific temperature management	–	Limited	–	–	–	Comprehensive
Integration of immature embryo rescue (IER) with speed breeding	–	Moderate	–	–	–	Comprehensive
Unified GASB framework	Partial	Partial	Partial	Partial	Partial	Comprehensive
Resource-based implementation strategy	–	–	–	–	–	Comprehensive
Standardised multi-environment validation framework	–	–	–	–	–	Comprehensive
Implementation roadmap with measurable milestones	–	–	–	–	Limited	Comprehensive

Although this review considers the broader *Gossypium* genus where relevant, the majority of experimentally validated studies currently available concern *G. hirsutum*. Therefore, discussions relating to *G. barbadense*, semi-wild accessions, landraces, and other *Gossypium* species are intended to identify knowledge gaps and future research opportunities rather than imply direct transferability of protocols across the entire genus.

## Flowering physiology and molecular targets for SB acceleration

### Evolutionary context: from obligate short-day plant to day-neutral crop

Most wild *Gossypium* species are reported to exhibit strong short-day flowering responses, although the magnitude of photoperiod sensitivity varies among species and accessions. Broad day-neutrality in cultivated *G. hirsutum* is thought to have evolved through prolonged artificial selection during adaptation to higher-latitude cultivation environments. Among the consequences of this evolutionary transition, three are particularly relevant to SB. First, the FLOWERING LOCUS C (*FLC*) gene — the central vernalisation-responsive repressor in *Arabidopsis thaliana* — has not been identified in the sequenced *Gossypium* genomes examined to date (Ren et al., 2017; Ma and Yan, 2022), suggesting that cotton lacks a classical vernalisation requirement, which may facilitate rapid generation cycling. Second, *G. hirsutum GhFT* peaks at 4–8 h of light exposure vs. the 16-h requirement for *AtFT* in *Arabidopsis* (Guo et al., 2015), suggesting that photoperiods approaching 22 h are likely to maximise *GhFT* expression under the reported experimental conditions, although responses may vary among cultivars and environmental conditions. The principal molecular targets and their potential strategies for integration into cotton speed breeding are summarized in **Table 3**. Third, semi-wild *G. hirsutum* races (e.g. subsp. *purpurascens*) and landrace accessions retain obligate short-day character (Abdurakhmonov et al., 2022; Kurbonov et al., 2024), while *G. barbadense* photoperiod sensitivity maps to the single major locus Gb_Ppd1 on chromosome D06 (Zhu et al., 2024). These distinctions help define the practical scope of protocols developed for elite day-neutral *G. hirsutum* and indicate where further modification may be required for diverse germplasm. Based on the available mechanistic evidence, an evidence-based multi-channel LED configuration for day-neutral G. hirsutum is summarized in **Table 4**.

**Table 3:** Priority molecular targets for SB integration in *Gossypium* spp.

Gene	Function	Direction	SB Strategy	Key evidence and SB relevance
*GhFT*	Florigen; systemic floral signal	Positive	22h/2h LED	Morning-phased expression peaks at 4–8h; 22h SB near-saturates induction (Guo et al., 2015)
*GhFKF1*	Blue-light CO stabiliser	Positive	450 nm LED	Hub gene; VIGS silencing delays 4.62 d; *GhFKF1*–GI–CO→*GhFT* axis (Pan et al., 2024)
*GhCOL3*	*GhFT* transcriptional repressor	Negative	CRISPR KO	VIGS silencing accelerates flowering; *bHLH38/BBX31* cascade (Hu et al., 2025)
*GhLUX1*	Evening complex; represses GhCOL1/*GhFT*	Negative	CRISPR/base edit	Silencing de-represses *GhFT*; circadian amplitude modulation (Hao et al., 2021)
*GhELF3*	Evening complex component	Negative	CRISPR/base edit	Co-regulates *GhLUX1*; silencing promotes early flowering (Hao et al., 2021)
*GhSOC1*	Floral integrator; 6 paralogs	Positive	Allele mining/OE	Convergence of GA, age, photoperiod; ChIP binding at *GhMADS41/42* (Ma and Yan, 2022)
*GhCAL*	Floral meristem identity	Positive	Stage marker for floral transition	3TLS (early) vs 5TLS (late): molecular marker of floral transition and earliness (Cheng et al., 2020)
*GhTFL1*	Floral repressor; architecture	Negative	Base editing	Base-editing of *GhTFL1* defines ideal plant architecture (Wang et al., 2024)
*GhAGL8/GhJAZ1*	Photoperiod QTL; D03+D08	Mixed	KASP/MABC	Fine-mapped loci; *GhJAZ1* links JA to flowering time (Zhang et al., 2025b)
*Gb_Ppd1*	*G. barbadense* SD locus; D06	Negative	KASP/CRISPR	Single-locus photoperiod control; tractable target for day-neutral ELS cotton (Zhu et al., 2024)

**Table 4:** Evidence-based multi-channel LED recipe for *G. hirsutum* SB (day-neutral elite cultivars).

Channel	Peak (nm/K)	Relative photon contribution (%)	Photoreceptor target	Mechanistic evidence
Red	660 nm	50–65%	PSII; phyB→Pfr	Primary photosynthesis driver; R:FR balance governs phyB activity state (Li et al., 2025; Valverde et al., 2004)
Blue	450 nm	15–25%	*cry1/cry2; GhFKF1; phot1/2*	CO stabilisation + *GhFT* induction; stomatal aperture; VIGS delay 4.62 d (Pan et al., 2024; Liu et al., 2016)
Far-red	730 nm	8–15%	phyB→Pr; phyA FR-HIR	R:FR 0.5–1.2; removes phyB-mediated CO repression; >15% induces shade avoidance (Blinkov et al., 2025)
Near-UV	395 nm	3–5%	UVR8; flavonoid synthesis	Flavonoid pathway highly enriched under LD in *G. hirsutum*; auxin transport modulation (Zhang et al., 2025)
Warm-white	3000–3500 K	Background	Broad PAR continuum	Red-enriched baseline; 3500 K advances wheat anthesis 4 d vs 4500 K at equal PPFD (Li et al., 2025)
Cool-white	6000–6500 K	Background	Broad PAR continuum	Blue-enriched continuum; compact morphology; supports cry/FKF1 activation

Recommended: PPFD 500–800 µmol m-2 s-1 | R:FR 0.5–1.2 | 22h/2h photoperiod (day-neutral) or staged 16h→10h (SD-sensitive) | DLI 39.6–63.4 mol m-2 d-1 | 2h dark minimum for PSII repair | Active air circulation 0.3–0.5 m s-1.

*Relative photon contribution (%)* refers to the proportion of the total emitted photon flux contributed by each spectral channel. The 395 nm (near-UV) and 730 nm (far-red) wavelengths lie outside the conventional 400–700 nm photosynthetically active radiation (PAR) range and are therefore not reported as percentages of PAR.

## The CO/FT pathway and *GhFKF1*: from LED wavelength to gene expression

The molecular framework governing photoperiodic flowering in *Gossypium* is architecturally conserved with the *Arabidopsis* CO/FT module but contains cotton-specific hubs that directly link LED spectral channels to gene expression outputs. The most significant hub for SB is *GhFKF1* — a FLAVIN-BINDING KELCH REPEAT F-BOX 1 a blue-light receptor that responds strongly to blue light centred around 450 nm under the reported experimental conditions. Under long-day and blue-light conditions, *GhFKF1* forms a complex with GIGANTEA protein to stabilise CO and drive *GhFT* transcription (Pan et al., 2024). VIGS-mediated silencing of *GhFKF1* delayed flowering by 4.62 days, demonstrating that the 450-nm LED channel in the SB recipe is a direct molecular intervention on the CO/FT pathway, not merely a photosynthetic supplement. Three negative regulators constitute the primary CRISPR targets for creating intrinsically early-flowering germplasm: *GhCOL3* (VIGS silencing accelerates flowering via *bHLH/BBX31* suppression; Hu et al., 2025), *GhLUX1* and *GhELF3* (evening complex; silencing de-represses *GhCOL1/GhFT*; Hao et al., 2021). The recommended stage-specific temperature conditions and critical thermal limits for G. hirsutum speed breeding are summarized in **Table 5**. The integrated stage-specific nutrition, micronutrient, biostimulant, and biochemical monitoring strategy is summarized in **Table 6**.

**Table 5:** Stage-specific temperature protocol for *G. hirsutum* SB in controlled environments.

Stage	Duration	Day temp.	Night temp.	Critical limit	Physiological rationale
Germination	0–7 DAE	28–30°C	22–25°C	Max: 38°C	TKW centre; uniform emergence within 5–7 d; below 18°C increases pathogen susceptibility
Seedling establishment	7–21 DAE	28°C	20–22°C	Max: 35°C	Maximum leaf area expansion rate; avoidance of chilling-induced growth delay
Rapid vegetative growth	21 DAE – squaring	28°C	20–22°C	Max: 32°C	TKW optimum; phyB thermal reversion at 28°C reinforces far-red flowering promotion
Squaring and bud development	Squaring – anthesis	28–30°C	18–20°C	POLLEN MAX: 33°C	Tetrad-stage protection; moderate night differential reduces respiratory C drain (Loka and Oosterhuis, 2010)
Anthesis and fertilisation	DAF 0–3	26–28°C	18–20°C	CRITICAL: 33°C	Pollen tube elongation window 12–24 h post-anthesis; fertilisation impaired above 33°C (Saini et al., 2023)
Boll and embryo development	DAF 3–30	28–30°C	20–22°C	Max: 35°C; Min: 20°C	Immature embryos are typically collected at 25–30 DAP for embryo rescue; 27–30°C supports embryo development and cotyledon starch accumulation prior to *in vitro* culture.
IER *in vitro* culture	DAP 25–30	27°C constant	16h/8h regime	Range: 24–32°C	>90% cotyledon expansion by day 6 in AUH medium; temperature precision ±2°C critical (Wang et al., 2025)
Plantlet acclimatisation	7–14 days	24–28°C	20–22°C	RH: 90→65% gradient	Gradual stepdown over 7–10 days to prevent transplant shock

**Table 6:** Integrated nutrition protocol and biochemical monitoring for *G. hirsutum* SB.

Stage	Macronutrients	Key micronutrients	Biostimulant/hormone	Biochemical monitoring
Germination (0–7 DAE)	N:P:K 1:0.5:1; complete solution	Fe-EDTA 5 mg L-1; Zn 0.5 mg L-1	—	Germination rate; seedling uniformity; cotyledon expansion
Vegetative growth (7–21 DAE)	High N (1.5–2× baseline); full K	B 0.3; Zn 1.0; Mn 0.5 mg L-1	Protein hydrolysate 1–2 g L-1 (foliar); GA3–5 ppm if delayed	SPAD 45–55 weekly; interveinal chlorosis = Fe deficiency
Squaring → anthesis	Taper N; peak K; add Ca	B 0.5 mg L-1 foliar — pollen critical; Zn+Mn maintain	NAA 5 ppm (anti-abscission); GA3 5–15 ppm	SPAD; proline <150 µg g-1 FW baseline; MDA <0.40 nmol g-1 FW
Boll development (DAF 0–30)	Balanced N:K:Ca; avoid excess N	B+Zn+Fe maintain; K foliar at peak bloom	IAA 5 ppm at DAF 0–5 (anti-abscission)	MDA <0.60; SOD/CAT/POD; TSS 2.5–3.5 mg g-1 FW

### MADS-box integrators and GhCAL as a marker of floral transition

Downstream of the CO/FT pathway, floral transition in *Gossypium* converges on a MADS-box transcription factor network. *GhSOC1* paralogs (six identified) integrate signals from the photoperiod, gibberellin, and age pathways (Ma and Yan, 2022). *GhMADS42* accelerates flowering when overexpressed in *Arabidopsis* (Zhang et al., 2016). Of outstanding practical importance, *GhCAL* — identified through high-resolution stage-specific transcriptomics — marks the onset of flower bud differentiation at the third true leaf stage (3TLS) in early-maturing varieties versus 5TLS in late-maturing ones (Cheng et al., 2020). This 3TLS molecular threshold marks the onset of floral transition and reproductive competence in early-maturing cotton genotypes. However, it should not be interpreted as determining the timing of immature embryo rescue, which depends on embryo developmental age after fertilisation rather than floral transition.

## Light spectrum, PPFD, and photoperiod: from SB 1.0 to SB 3.0

### Technology progression and the three-objective optimisation problem

The evolution from first-generation HPS photoperiod extension (SB 1.0) through two-channel R+B LED systems (SB 2.0) to full multi-channel spectral control (SB 3.0; Kabade et al., 2025) represents progressively more precise resolution of a three-objective tension: simultaneously maximising photosynthetic DLI, activating the CO/FT pathway for early floral transition, and preventing shade-avoidance syndrome from excessive far-red. These objectives are partially antagonistic. SB 3.0 addresses this challenge by integrating multiple spectral channels that target complementary photoreceptor pathways based on the currently available evidence. However, comprehensive spectral-removal or factorial validation in cotton is still lacking. The principal in vitro biotechnology approaches and their roles in cotton speed-breeding integration are compared in **Table 7**.

**Table 7:** Comparative *in vitro* biotechnology protocols for cotton SB integration.

Technology	Optimal stage	Key medium	Critical parameter	Primary performance indicator	GASB role
Callus induction	5–7 d post-germination	MS + 0.1 mg L-1 2,4-D + 0.5 mg L-1 kin	Genotype responsiveness; 4–6 mo to T0	Callus induction frequency (60–95%; genotype-dependent)	CRISPR/NGT entry; hybrid rescue
Genetic transformation	Callus or protoplast	Agrobacterium or RNP delivery	Window position within PAM; off-target rate	Transformation efficiency (26–57%; GhBE3 system)	Precise allelic changes; transgene-free option (Qin et al., 2019)
Genome editing	Embryogenic callus or regenerable explants	Transformation medium appropriate for the editing platform	Transformation efficiency; editing specificity; off-target frequency	Reported editing efficiency (%)	Targeted genome modification; rapid trait improvement
Microspore culture	4–6 mm flower buds	Modified induction medium (representative formulation)	Late uninucleate stage; cold pretreatment; sterile culture conditions	Reported embryoid induction frequency	5 gen/yr; cycle 71–85 d (Wang et al., 2025)
Acclimatization	2–3 true leaves	Gradual ex vitro transfer	RH 90→65% stepdown over 7–10 d	Plant survival after acclimatization (%)	Establishment of regenerated plants for rapid generation advancement

The primary performance indicators listed in this table represent different biological and technical endpoints at successive stages of the regeneration pipeline and therefore should not be interpreted as directly comparable measures of overall protocol success. Furthermore, editing efficiency does not necessarily indicate the recovery of stable, non-chimeric, heritable edited plants, and embryoid induction or embryo germination does not necessarily result in fertile plants.

The proposed GASB molecular-marker pipeline, including marker levels, target traits, infrastructure requirements, and priorities, is summarized in **Table 8**. The four-channel architecture operates through distinct, non-overlapping molecular interfaces. 660 nm red drives Photosystem II electron transport and activates phyB to its Pfr form — the dominant photosynthetic input. 730 nm far-red converts phyB to inactive Pr through phyA far-red high-irradiance responses, removing CO repression and promoting *GhFT* accumulation. The R:FR ratio (optimally 0.5–1.2 for cotton SB) governs the phyB activity equilibrium. 450 nm blue activates *cry1/cry2* and *GhFKF1* through a phyB-independent pathway, providing a second route to CO stabilisation and *GhFT* transcription (Pan et al., 2024; Liu et al., 2016). 395 nm near-UV activates UVR8 and the flavonoid biosynthesis pathway — one of the most significantly enriched metabolic modules under long-day conditions in *G. hirsutum* (Zhang et al., 2025). Current evidence suggests that each spectral channel contributes distinct physiological functions within the SB system. However, the relative contribution of individual wavelengths and the minimum effective spectral combination remain to be determined through systematic spectral-removal or full factorial experiments in cotton. The major seed-to-seed speed-breeding protocols currently applicable to *Gossypium spp*. are compared in **Table 9**.

**Table 8:** GASB molecular marker pipeline: tools, traits, and priorities.

Tool/level	Marker(s)/method	Target trait(s)	Infrastructure	Performance and notes
L1: SSR	BNL0663, BNL1694, BNL3347, BNL3436, BNL3601, NAU1042, NAU2173, NAU2508	Early maturity; diversity	PCR + gel	Validated across Upland cotton; BNL-3601 (170 bp) associated with early maturity (Hinze et al., 2017)
L1: SSR	JESPR095 + BNL1421	Salt-tolerant donor ID	PCR + gel	JESPR095 (110 bp); BNL1421 (235/180 bp biallelic); salt-tolerant donor identification (Tussipkan et al., 2024)
L2: KASP	D06-5486215	Heat tolerance (pollen)	RT-PCR reader	>85% selection accuracy; validated in 308 accessions/2 seasons (Zhao et al., 2025)
L2: KASP	22079 + 22089 (Hap8)	Drought tolerance	RT-PCR reader	Hap8 allele: 100% accuracy in 302 accessions; GWAS-derived (Liu et al., 2025)
L2: KASP	GhAGL8/GhJAZ1 flanking SNPs	Photoperiod-neutral phenology	RT-PCR reader	D03+D08 fine-mapped; pre-screen for day-neutral SB compatibility (Zhang et al., 2025b)
L3: SNP array	CottonSNP63K (45K SNPs)	All agronomic — GS training	Array scanner + bioinformatics	GS training platform; r = 0.64–0.76 for fibre traits (Li et al., 2022; Hulse-Kemp et al., 2015)
L3: GBS	NGS-based; 15–50K SNPs	GWAS + GS training	NGS + HPC pipeline	Cost-effective; 2–4 wk turnaround compatible with SB timelines (Raza et al., 2024)
L3: FibreGGP	RNA-seq + GCN; ~30 SNPs/trait	Fibre length/strength	NGS + bioinformatics	Biology-informed Bayesian GS; outperformed Bayes-C in the original study population; reported 10–20× reduction in genotyping requirements (Khalilisamani et al., 2024)

**Table 9:** Comparative seed-to-seed SB protocols for *Gossypium* spp.

Protocol	Target germplasm	Gen/year	Day temp.	Photoperiod	Key elements
LED-only SB (no IER)	*G. hirsutum elite, day-neutral*	4	28/18°C	22h/2h	Multi-channel LED; stage-specific nutrition; sowing to boll ~100–110 d; no tissue culture required; entry-level GASB
LED + IER	*G. hirsutum elite*	5	28/18°C	22h/2h	As above + embryo excision at 25–30 DAP; AUH liquid at 27°C/16h; cotyledon expansion by d6; cycle 71–85 d (Wang et al., 2025)
Staged photoperiod (*G. hirsutum* landrace)	*SD-sensitive landraces*	3	28/18°C	16h→10h at 3TLS	LD phase builds biomass; SD switch at 3TLS (GhCAL threshold); timing requires genotype-specific calibration
Staged photoperiod (*G. barbadense)*	*ELS cotton landraces*	2–3	28/18°C	16h→10h	Gb_Ppd1 (D06) sensitivity requires SD; KASP pre-screening for reduced-sensitivity alleles recommended
SB-MABC	*Elite × donor*	4 BC gen/~1.5 yr	28/18°C	22h/2h	BC1→BC4F3 in 1.5 yr demonstrated (Wang et al., 2025); KASP foreground MAS each generation; background by SNP array
SB-DH (microspore)	*Transformation-responsive*	1 gen to DH	28/18°C	22h/2h to F1	4–6 mm bud collection; 4°C 24–48h pre-treatment; K-based pH 7.0 medium; colchicine doubling; ~3–4 mo to DH

## Daily light integral and PPFD quantification

The daily light integral (DLI) = PPFD (µmol m-2 s-1) × photoperiod (h) × 3600/1,000,000 integrates intensity and duration into the metric most predictive of biomass accumulation rate. In spring wheat SB, optimal DLI for seedling development was approximately 39.6 mol m-2 d-1 at 500 µmol m-2 s-1/22 h, with heading and flowering occurring 5.9 and 7.5 days earlier than at the lowest tested DLI (Zhong et al., 2026). Increasing PPFD to 700 µmol m-2 s-1 advanced wheat anthesis by 3–10 days, with highest lint yield also at 700 µmol m-2 s-1 (Li et al., 2025). Cotton’s substantially higher light saturation point (LSP ~800–1200 µmol m-2 s-1) compared with cereals means that photosynthetic gains from elevated PPFD extend to higher intensities before saturation, supporting a recommended range of 500–800 µmol m-2 s-1 for cotton SB. The extended PAR range (400–750 nm, ePAR) contributes photosynthetic electrons through the Emerson enhancement effect on PSI (Zhen and Bugbee, 2020), meaning broad-spectrum SB lamps deliver higher effective DLI per unit of measured 400–700 nm PAR than narrow-band R+B systems. The major biological, technical, operational, and implementation challenges of GASB, together with their causes, severity, and potential mitigation strategies, are summarized in **Table 10**.

**Table 10:** Structured challenge matrix for GASB implementation in *Gossypium* spp.

Domain	Challenge	Root cause	Severity	Evidence-based mitigation
Biological	Allotetraploid genome redundancy	38.2% of genes show PAV (Huang et al., 2021); homeolog compensation masks single-locus CRISPR edits	High	Multiplex CRISPR (Wang et al., 2017); base editing (Qin et al., 2019; Wang et al., 2024); prime editing for allelic precision (Sheri et al., 2025)
Biological	G×L interaction: SD-sensitive and landrace germplasm	*GhFT* suppression under 22h LD; *Gb_Ppd1* (D06) absent from elite backgrounds	High	Staged photoperiod (16h→10h at 3TLS); KASP pre-screening at *GhAGL8/GhJAZ1 and Gb_Ppd1* loci; genotype-specific calibration (Zhu et al., 2024)
Biological	SB-to-field correlation: unvalidated in cotton	GEI >79% of yield variance (Deshmukh et al., 2023); IER lines selected under artificial conditions diverging from field	High	Mandatory F4–F5 multi-environment trials; genomic G×E models (Crossa et al., 2021); systematic publication of SB vs field r2 — highest priority knowledge gap
Biological	Narrow genetic base in elite germplasm	Domestication bottleneck: 30–40% less diverse than wild/landrace pools (Huang et al., 2021)	Medium	Strategic landrace × elite crosses each programme cycle; PAV screening of breeding panels; diverse donor integration via SB-MABC
Biological	Epigenetic stability across rapid SB generations	Constant high-PPFD/temperature may cause directional methylation change at stress-adaptive loci	Low–Med	Whole-bisulfite sequencing pilot across ≥3 SB generations at *GhFT* and stress-response promoters
Technical	Pollen safety ceiling: thermally invisible	Heat damage precedes visible symptoms; silent generation failure (Masoomi-Aladizgeh et al., 2021)	High	Canopy IR thermometry continuously logged at squaring; 0.3–0.5 m s-1 air circulation; hard 33 °C operational ceiling
Technical	IER scalability and contamination risk	Sterile microsurgical facility required; contamination failure 15–30% per batch	High	LED-only 4-gen/yr as interim target; IER for well-equipped facilities only; rigorous 0.1% HgCl2 sterilisation protocol
Technical	Fibre quality phenotyping throughput	HVI testing requires mature bolls; incompatible with seedling-stage selection timing	Medium	GS-based GEBV prediction reduces the need for early-generation fibre-quality phenotyping but still depends on high-quality multi-environment phenotypic data for model development, calibration, validation, and periodic updating (Li et al., 2022; Rolland et al., 2022; Renó et al., 2024).
Technical	Bioinformatics infrastructure	HPC + GPU + NAS required for GBS, GWAS, and GS model training	High	FibreGGP (~30 SNPs/trait) was reported to reduce computational demand in the original implementation; cloud-based GBS pipelines may provide an interim solution.
Knowledge	G×L interaction data for non-elite germplasm	Published SB data from only 2–3 Chinese elite varieties; <1% of *Gossypium* germplasm characterised	High	Multi-genotype G×L study (≥20 accessions × 4 LED recipes) is the single highest-priority experimental study for cotton SB adoption
Knowledge	*G. barbadense* SB protocol	No published SB protocol achieving ≥3 gen/yr for *G. barbadense* as of this review	High	Staged-photoperiod + *Gb_Ppd1* KASP pre-screening; long-term: CRISPR of *Gb_Ppd1* for day-neutral ELS (Zhu et al., 2024)
Institutional	SVT regulatory timeline	3–5 year mandatory field testing is legally independent of SB upstream speed	Medium	Evidence-based policy advocacy for streamlined NGT pathway; isogenic-line approach for near-identical GASB lines
Institutional	CRISPR/GMO regulatory classification	CRISPR with T-DNA classified as GMO in Uzbekistan, Pakistan, and other major producers	High	Transgene-free RNP or base-editing delivery; NGT legal distinction from GMO; regulatory reform parallel to technical development (Sheri et al., 2025)
Institutional	Human capital deficit	GASB requires simultaneous competence in physiology, molecular biology, tissue culture, bioinformatics, and agronomy	Medium	Graduate training; international collaboration (CSIRO, Hebei Agr. Univ., JIC); 5–10 year sustained capacity investment

### Photoperiod management for day-neutral versus short-day germplasm

For day-neutral elite *G. hirsutum*, a constant 22h/2h photoperiod is standard. Under optimised spectral conditions, this produces first flower buds at 19–21 DAE and anthesis at ~45–46 DAE (Wang et al., 2025). Long-day conditions advance budding and flowering by ~20 and ~17 days vs. short-day conditions, with differential gene expression enriched in flavonoid biosynthesis, jasmonate signalling, and α-linolenic acid metabolism pathways (Zhang et al., 2025). For *G. barbadense* and semi-wild *G. hirsutum* with residual short-day sensitivity, a staged-photoperiod approach is required: 16–18h LD to build vegetative biomass, then switch to 10–12h SD at the 3TLS threshold (the *GhCAL* developmental checkpoint) to trigger floral initiation (Jähne et al., 2020; Zhu et al., 2024). The timing of the LD→SD switch requires genotype-specific calibration and represents the most important experimental knowledge gap for programmes deploying SB with diverse germplasm. Illustrative investment requirements, expected benefits, and economic implications across progressive GASB implementation levels are summarized in **Table 11**.

**Table 11:** Illustrative cost–benefit considerations for progressive GASB implementation.

Resource level	Major investment	Primary benefit	Expected economic impact
Basic	Photoperiod extension, SSD, SSR/KASP	Faster generation advancement and earlier elimination of unsuitable lines	Low capital requirement; moderate reduction in cycle time and field-generation costs
Intermediate	Controlled-environment facility, targeted SNP/KASP genotyping, structured phenotyping	Faster advancement combined with improved selection accuracy	Moderate capital requirement; lower cost per selected line and improved operational efficiency
Advanced	Genome-wide genotyping, high-throughput phenotyping, AI tools and genomic prediction	Maximum annual genetic gain and earlier cultivar nomination	High initial investment; greatest potential long-term return when used at sufficient programme scale

## Genotype-specific calibration of staged photoperiod protocols

**Figure 1:**
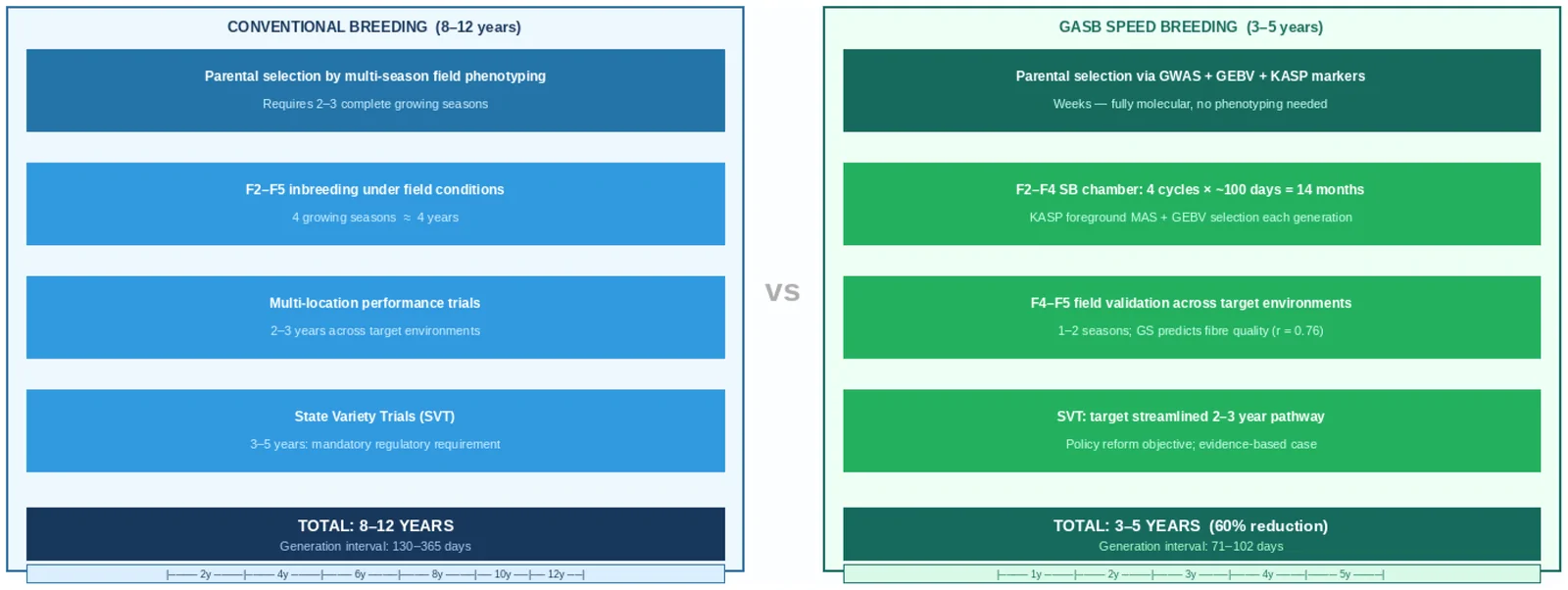
Comparative workflow: conventional cotton breeding (left) versus the GASB pipeline (right). GASB compresses the total variety development timeline from 8–12 years to 3–5 years by reducing each phase: parental selection (weeks vs. 2–3 seasons), inbreeding (14 months vs. 4 years), field validation (1–2 vs. 2–3 seasons), and targeting a streamlined SVT pathway. GEBV, Genomic Estimated Breeding Value; GS, Genomic Selection; KASP, Kompetitive Allele-Specific PCR; SVT, State Variety Trials.

Because residual photoperiod sensitivity varies considerably among *Gossypium* species, landraces, and elite cultivars, a universal long-day to short-day transition is unlikely to maximise flowering across diverse germplasm. Instead, staged photoperiod protocols should be calibrated for each breeding population before routine deployment (**Figure 1**). A practical calibration strategy would involve evaluating representative genotypes under several LD→SD transition points while recording days to flower initiation, first flowering, boll set, seed production, and total seed-to-seed generation time. These data can be analysed using mixed-effect models to estimate genotype × photoperiod interactions and identify the transition stage that minimizes generation interval without reducing reproductive success. Once calibrated for a representative diversity panel, closely related breeding populations may use the same protocol with only minor adjustments. Such genotype-specific optimisation is expected to become increasingly important when speed breeding is extended beyond elite day-neutral *G. hirsutum* into *G. barbadense*, landraces, wild relatives, and interspecific breeding populations.

## Thermal biology and stage-specific temperature optimisation

### The thermal kinetic window and evidence-based temperature guidance for pollen protection

**Figure 2:**
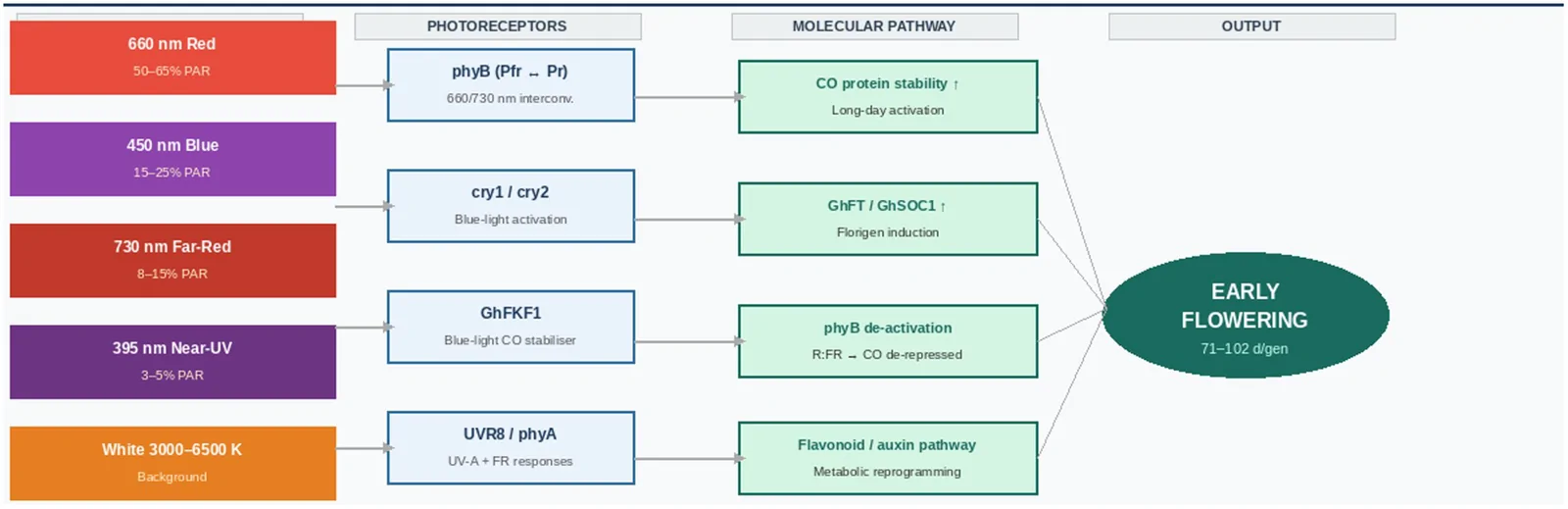
LED spectral channels, photoreceptor targets, and downstream molecular consequences in the cotton SB flowering pathway. The four spectral channels target complementary photoreceptor pathways based on the currently available evidence, converging on the regulation of flowering through distinct molecular mechanisms. CO, CONSTANS; GhFT, FLOWERING LOCUS T homolog; GhFKF1, FLAVIN-BINDING KELCH REPEAT F-BOX 1; phyB, phytochrome B; UVR8, UV RESISTANCE LOCUS 8.

**Figure 3:**
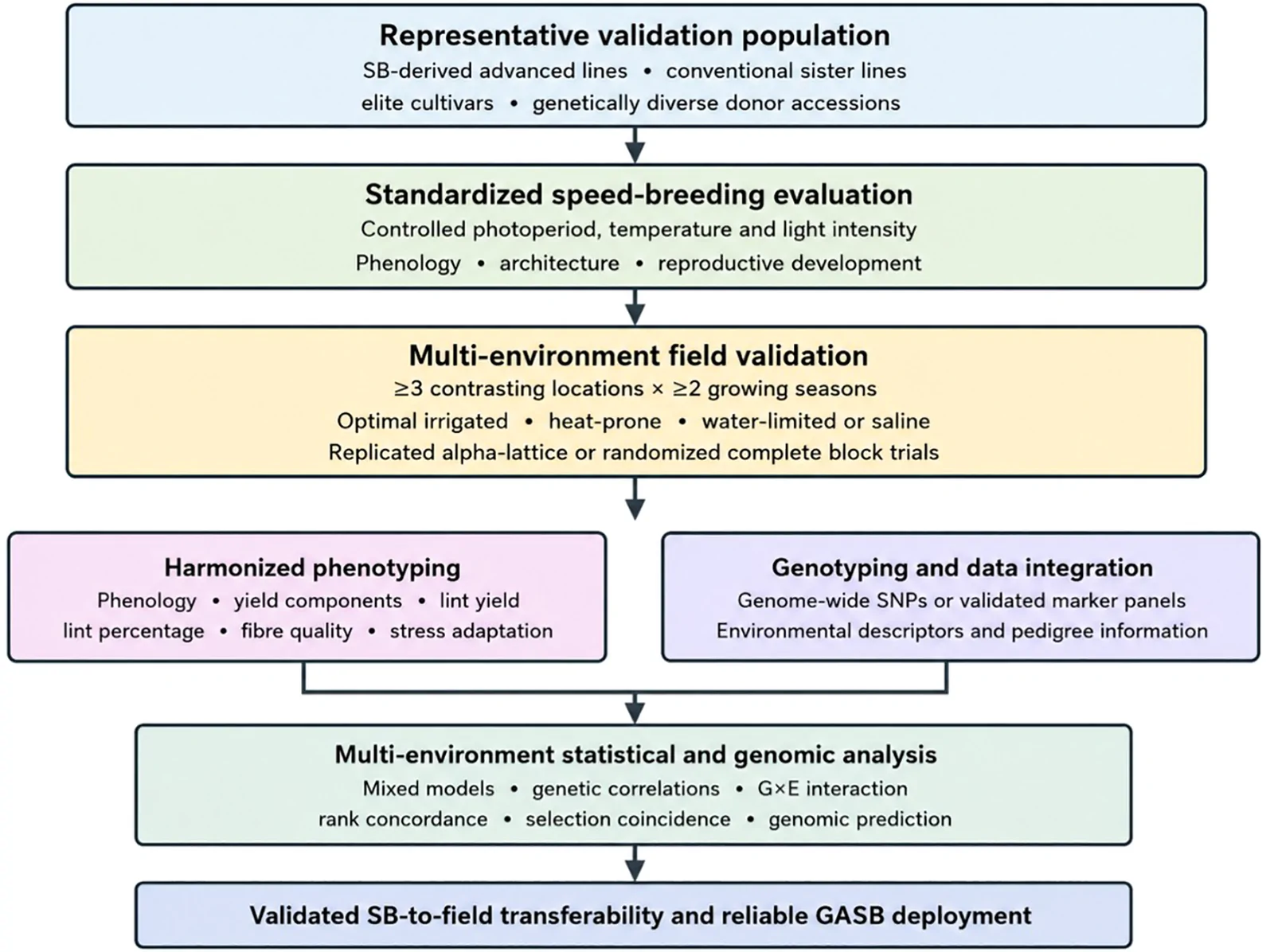
Proposed multi-environment validation framework for genomics-assisted speed breeding (GASB) in cotton. The framework illustrates a standardised workflow for validating the transferability of SB-derived selection decisions from controlled environments to diverse field conditions. Representative breeding populations are evaluated under standardised SB conditions and subsequently tested across contrasting field environments using harmonised phenotyping, genome-wide genotyping, and integrated multi-environment statistical analyses. The resulting SB-to-field genetic correlations, genotype-rank concordance, selection coincidence, and genomic prediction accuracy provide an evidence-based foundation for reliable implementation of GASB in cotton breeding programmes.

The thermal kinetic window (TKW) of *G. hirsutum* (23.5–32°C; optimum 28°C; Burke et al., 1988) defines the metabolic throughput envelope for vegetative SB growth (**Figure 2**). The reproductive thermal response, however, operates through a distinct physiological mechanism and indicates that temperatures above approximately 33°C are associated with an increased risk of pollen damage under many experimental conditions (**Figure 3**). Tetrad-stage pollen — at 4–6 mm bud length — is the most thermally vulnerable developmental point: exposure to 40/30°C for 5 days reduces boll production by 65% and pollen grain size by 36% without any visible vegetative symptoms (Masoomi-Aladizgeh et al., 2021). Pollen germination declines steeply above 33°C and approaches zero above 39°C (Liu et al., 2014). Combined elevated day/night temperatures (37/26°C) reduce seed and lint yield by 50–60% (Mishra et al., 2017; Saini et al., 2023). A single undetected excursion above 35°C during the tetrad stage can therefore silently negate an entire generation advance — highlighting the importance of canopy infrared thermometry for monitoring reproductive-stage temperature under high-light conditions (PPFD >500 µmol m^-^² s^-^¹).

### Temperature × light synergism: phytochrome thermosensory function

Temperature and light interact through phytochrome thermosensory function with direct implications for SB protocol design. At 28°C (the standard SB setpoint), phyB Pfr→Pr thermal reversion accelerates compared with its rate at 20°C under identical red-light illumination (Jung et al., 2016). This thermally mediated phyB inactivation provides a flowering-promoting mechanism independent of far-red supplementation, so that the 28°C standard achieves a three-way optimisation: maximum photosynthetic throughput (TKW centre), partial phyB de-repression through thermal reversion, and enhanced *GhFT* amplitude through temperature-clock interactions (Luo et al., 2025). A diurnal differential of 28°C day/18–20°C night — rather than constant 28°C — further reduces nocturnal respiratory carbohydrate drain from developing bolls (Loka and Oosterhuis, 2010), improving both seed set and generation advance efficiency. Prasad et al. (2024) confirmed that such thermoperiod protocols consistently optimise SB outputs across warm-season crops.

At PPFD >600 µmol m-2 s-1, LED radiative heat loading elevates canopy temperature 2–4°C above chamber air temperature. Active air circulation at 0.3–0.5 m s-1 and continuous canopy infrared thermometry during squaring are non-negotiable in any cotton SB facility. The 33°C pollen ceiling is a hard operational limit — not a guideline.

## Nutritional physiology, hormones, and biochemical monitoring

### Accelerated nutrient demand under high-PPFD SB conditions

Under high-PPFD SB conditions, nutrient demand per unit time is elevated proportionally to photosynthetic rate, and deficiencies that develop over 8–12 weeks in field conditions manifest within 3–4 weeks in SB chambers. This temporal compression requires proactive, stage-specific nutritional management. Nitrogen is the dominant macronutrient during vegetative SB, with peak demand coinciding with first flower bud appearance and N accumulation in reproductive organs being quantitatively more important for yield than in vegetative tissues (Chen et al., 2020; Ma et al., 2024). Cotton is among the most K-demanding agronomic crops: K deficiency reduces stomatal aperture, impairs phloem sucrose loading to reproductive sinks, and depletes carbohydrate supply to developing bolls (Bumguardner et al., 2024; Cai et al., 2016).

### Critical micronutrients for SB reproductive success

Among micronutrients, boron is the single most critical for SB reproductive success. Its essential roles in pollen tube elongation and boll set through borate ester cross-linking of cell wall rhamnogalacturonan II mean that B deficiency of even short duration during the 4–6 mm tetrad stage can cause widespread boll abortion without visible vegetative symptoms (Ahmed et al., 2025; Naeem et al., 2018). Zinc deficiency impairs IAA biosynthesis through its role in tryptophan synthesis, reducing pollen viability and increasing premature boll abscission (Li et al., 2022). Iron deficiency causes interveinal chlorosis that directly reduces photosynthetic rate under high-PPFD conditions, while manganese deficiency accelerates PSII photoinhibition at near-maximum throughput by impairing the oxygen-evolving complex (Ahmed et al., 2025). The B–Zn synergistic interaction in cotton reproductive tissue — where combined application improves Fe and Mn transport to bolls — underscores the need for balanced multi-element micronutrient management.

### Biostimulants, plant hormones, and anti-abscission management

Plant-derived protein hydrolysates have been reported to promote reproductive development through several complementary mechanisms, although their effectiveness may vary depending on genotype, formulation, application rate, and environmental conditions: (i) pre-formed amino acids bypass the energetically expensive GOGAT nitrogen assimilation pathway during peak reproductive metabolic demand; (ii) polyamines (putrescine, spermidine, spermine) exert anti-abscission effects at floral pedicels through interactions with the ethylene pathway (Podlesáková et al., 2019; Huang et al., 2023); and (iii) tryptophan provides substrate for endogenous IAA biosynthesis that maintains the IAA:ethylene ratio above the abscission threshold at pedicel abscission zones (Dong et al., 2019; Rouphael et al., 2015; Colla et al., 2015). Gibberellin application (Published studies have reported that GA_3_ applications of approximately 5–15 ppm are effective for promoting flowering under specific experimental conditions. However, the optimal concentration may vary among genotypes and cultivation conditions) promotes the floral transition through DELLA degradation and de-repression of *GhSOC1/GhFT* expression (Zhang et al., 2025). Exogenous IAA application at approximately 5 ppm has been reported to improve reproductive performance under the experimental conditions described by Dong et al. (2019). However, the optimal concentration may vary among genotypes and cultivation systems.

## Biochemical stress monitoring in SB programmes

In SB systems where each generation represents a significant resource investment, early-warning biochemical markers are of high practical value. SPAD values below 40 units indicate N or micronutrient deficiency requiring immediate correction; values of 45–55 represent the optimal range under well-managed SB conditions (Wu et al., 2022). Leaf proline above 300 µg g-1 FW indicates osmotic stress onset that will impair pollen development (Characterizing, 2025). Malondialdehyde (MDA) above 0.60 nmol g-1 FW indicates significant oxidative membrane damage; simultaneous elevation of both proline and MDA signals that stress has exceeded cellular protective capacity, at which point harvesting the current generation and restarting is preferable to waiting for visible symptoms. Reference antioxidant enzyme activities under optimal SB conditions are SOD ~300–500 U g-1 FW, POD ~350–450 U g-1 FW, and CAT ~250–350 U g-1 FW (Ahmed et al., 2025).

## *In vitro* biotechnology and immature embryo rescue

### Callus culture as the CRISPR transformation gateway

Efficient *in vitro* regeneration performs two distinct functions in GASB: it provides the Agrobacterium-mediated transformation pathway for CRISPR and NGT editing, and enables rescue of inter-specific hybrids that abort in planta. Cotton is notoriously recalcitrant to *in vitro* regeneration — efficiency is highly genotype-dependent, with responsive lines sharing high endogenous cytokinin content in hypocotyl tissue that facilitates the callus-to-somatic-embryo transition (Thangaraj et al., 2025; Raza et al., 2024; Khojaqulova et al., 2025). Standard protocols require kanamycin-selection of transformants on MS medium with 2,4-D and kinetin, generating T0 transgenic plants within 4–6 months. This timeline is incompressible within a single SB generation, meaning that CRISPR editing and SB cannot be combined in the same generation cycle.

Several NGT advances are actively addressing the recalcitrance and transformation bottleneck. Base editing in cotton (*GhBE3* system; Qin et al., 2019) enables precise C→T substitutions at target sites with efficiency of 26–57%, without DNA double-strand breaks. Precise fine-tuning of GhTFL1 — a key flowering-time and architecture regulator — by base-editing tools defined an ideal compact cotton plant architecture (Wang et al., 2024). Multiplex CRISPR editing of multiple homeologous loci simultaneously, using the high-efficiency system developed by Wang et al. (2017), addresses the allotetraploid redundancy challenge that renders single-locus edits of flowering-time suppressors phenotypically silent. Ribonucleoprotein (RNP) delivery for DNA-free editing is advancing as a transgene-free route compatible with regulatory frameworks prohibiting transgenic cotton in Uzbekistan and several other major producing countries (Sheri et al., 2025).

### Microspore culture and stage-specific operational management

Microspore or anther culture has the potential to replace several generations of selfing through doubled-haploid (DH) production, where efficient DH recovery systems are available. However, in cotton, practical implementation remains constrained by strong genotype dependence and major bottlenecks in embryoid induction, plant regeneration, chromosome doubling, and recovery of stable fertile DH lines (Raza et al., 2024). In *G. hirsutum*, the androgenically responsive stage corresponds to the late uninucleate to early binucleate microspore transition at bud lengths of 4–6 mm. Maintaining microspore viability during isolation requires careful attention to medium osmolarity and ionic composition: ammonium, calcium, and magnesium salts at standard concentrations used for other species are lethal to cotton microspores, and an organically buffered pH of 7.0 with potassium as the primary cation is critical for survival (Barrow, 1986). Cold pre-treatment at 4°C for 24–48 h releases microspores from normal gametophytic development into the totipotency pathway through epigenetic stress-mediated reprogramming. Published studies have reported chromosome doubling efficiencies of approximately 70–80% following colchicine treatment under specific experimental conditions. However, successful chromosome doubling does not necessarily result in stable fertile DH plants.

From an operational perspective, the optimal developmental stage for microspore isolation coincides with the period of highest pollen sensitivity to elevated temperature, requiring careful scheduling of environmental control and sampling activities within speed-breeding facilities (Masoomi-Aladizgeh et al., 2021). This operational overlap highlights the importance of carefully coordinating microspore isolation and temperature management when both activities are conducted within the same breeding facility. Consequently, careful management during this developmental stage may improve the likelihood of successful pollen preservation, microspore isolation, and coordinated breeding operations, although successful recovery of stable fertile DH plants remains dependent on successful embryoid induction, plant regeneration, chromosome doubling, and fertility restoration.

## Immature embryo rescue: maximum generation rate

IER compresses the seed-to-seed cycle below in planta seed maturation time by excising immature embryos at 25–30 DAP and culturing them at 27°C in AUH liquid medium (half-strength MS + 3% sucrose + 0.01 mg L-1 BAP). Wang et al. (2025) achieved >90% success in producing transplantable plantlets with fully expanded green cotyledons within 6 days, enabling the five-generation-per-year cycle. Four parameters critically control IER success: harvest timing (23–33 DAP window; outside this range germination fails or enters dormancy); culture temperature precision (27°C ±2°C; deviations reduce germination by 15–30%); medium composition (AUH liquid outperforms solid Murashige–Skoog and Gamborg B5); and surface sterilisation (0.1% HgCl2 for 8 min after 70% ethanol prewash). Success rates exceed 85% in responsive *G. hirsutum* genotypes but fall below 50% in many landrace accessions, limiting applicability to transformation-responsive elite backgrounds.

## Molecular markers, genomic selection, and the GASB hierarchy

### Three deployable resolution levels

GASB operates at three nested resolution levels, each independently deployable as capacity develops:

Level 1 — SSR/KASP foreground MAS: single-locus seedling-stage selection using PCR and gel or fluorescent plate reader. Immediate entry point for any programme transitioning to SB-integrated selection. SSR-based MAS using marker BNL1604 has been validated for fibre quality improvement in naturally coloured G. *hirsutum* breeding programmes in Uzbekistan (Kurbonov et al., 2025a).

Level 2 — KASP multi-trait panels: GWAS-validated assays for heat tolerance (D06–5486215 locus; reported accuracy >85% in the original validation population; Zhao et al., 2025), drought tolerance (22709 + 22089, Hap8 allele; reported 100% accuracy in the original study population; Liu et al., 2025), and salt tolerance (Tussipkan et al., 2024). A panel of 20–30 validated KASP assays, requiring only a real-time PCR instrument, constitutes the operational core of a functioning GASB programme. Because marker performance depends on genetic background and population structure, these reported accuracies should not be interpreted as universally applicable and should be independently validated before implementation in other breeding populations.

Level 3 — GBS-based genomic selection: genome-wide SNP data in GBLUP, Bayesian, or transcriptomics-enhanced GS models for GEBV estimation of complex quantitative traits at the seedling stage. Li et al. (2022) reported cross-validated prediction accuracies of 0.76 (fibre length), 0.65 (fibre strength), and 0.64 (lint yield) in their original germplasm population and validation design. Prediction accuracy may vary substantially among breeding populations depending on training-population relatedness, marker density, phenotypic quality, trait heritability, and validation strategy (Khalilisamani et al., 2024), which incorporates RNA-seq-derived gene co-expression network (GCN) priors into a Bayesian GS framework, reduces the informative SNP set to ~30 per fibre quality trait — a 10–20× cost reduction that makes biology-informed GS accessible to programmes with limited sequencing infrastructure.

### Genomic resources and QTL mapping for SB-relevant traits

The cotton pan-genome assembled from 1,961 accessions (Huang et al., 2021) identified 32,569 non-reference genes, with 38.2% of all genes showing presence-absence variation (PAV) including 124 PAVs linked to fibre quality and yield loci inaccessible through single-reference GWAS. A recent F_2_/F_3_ QTL study identified 102 loci for fibre quality and early maturity, including qFL-D13–1 contributing 3.94–11.39% of fibre length phenotypic variance across generations (Jia et al., 2025). Fine mapping of *GhAGL8* and *GhJAZ1* on chromosomes D03/D08 as candidates for photoperiod-sensitive flowering (Zhang et al., 2025b) provides KASP targets for pre-screening populations for day-neutral SB compatibility — critical for programmes incorporating semi-wild donors. Deep-learning-assisted GWAS for fruit branch angle in 355 upland cotton accessions (Li et al., 2024) illustrates the growing integration of AI phenotyping tools with molecular genetics in cotton breeding.

### SB-to-field correlation: the most urgent knowledge gap

A fundamental unresolved question for GASB implementation is the extent to which selection decisions made in SB chambers translate to field performance across target environments. Blinkov et al. (2025) provided a systematic review of SB protocols and applications, listing phenotypic traits with documented high correlation between controlled-environment and field conditions in cereals — but equivalent data for cotton do not exist in the peer-reviewed literature. Genotype × environment interaction (GEI) contributes more than 79% of total yield variance in upland cotton across multi-location trials (Deshmukh et al., 2023), and SB-chamber conditions differ fundamentally from any field environment. GS models that explicitly incorporate GEI effects — such as the genomic G×E model structures evaluated by Crossa et al. (2021) — improve prediction accuracy across environments and should be the default modelling framework for cotton GASB programmes where the training population spans both SB-chamber and multi-location field phenotyping. Systematic publication of SB-vs-field r2 values for cotton traits is therefore the single highest-priority knowledge gap for field adoption of cotton GASB.

## Proposed multi-environment validation framework for genomics-assisted speed breeding in cotton

Despite the substantial reduction in generation interval achieved through speed breeding (SB), its routine deployment in cotton breeding ultimately depends on demonstrating that selection decisions made under controlled environments remain predictive of performance under field conditions. At present, no standardised framework exists for evaluating the transferability of SB-derived phenotypic and genomic selection to diverse production environments. We therefore propose a practical multi-environment validation framework that could serve as a reference for future cotton genomics-assisted speed breeding (GASB) studies.

The validation population should comprise representative *Gossypium hirsutum* germplasm, including SB-derived advanced breeding lines together with conventionally advanced sister lines originating from the same parental crosses, supplemented by elite commercial cultivars and genetically diverse donor accessions. Comparison of SB-derived and conventionally advanced sister lines is particularly important because it enables the effect of accelerated generation advancement to be distinguished from differences arising from genetic background or selection history.

Validation should integrate both controlled-environment and field evaluation. The same breeding population should first be characterised under the standardised SB conditions adopted by the breeding programme and subsequently evaluated across multiple contrasting field environments representing the major cotton production systems. Whenever possible, field testing should include at least three geographically distinct locations encompassing favourable irrigated conditions and one or more target stress environments, such as heat- or water-limited production systems, over at least two growing seasons. Multi-location trials should preferably employ alpha-lattice or randomised complete block designs with appropriate biological replication to ensure reliable estimation of environmental and genotype × environment (G×E) effects.

A harmonised phenotyping protocol should be applied across all environments. Core observations should include flowering and maturity, plant architecture, yield components, lint yield, lint percentage, and the principal fibre-quality traits, including upper-half mean length, fibre strength, micronaire, and fibre uniformity. Where stress environments are included, additional physiological or stress-adaptive traits relevant to the target production system may also be incorporated. Because some economically important traits cannot be measured directly during the accelerated SB cycle, validation should distinguish between traits assessed directly under both environments and those predicted through molecular or genomic information.

Phenotypic data should be analysed using multi-environment mixed models to estimate variance components, heritability, genotype × environment interactions, and genotype ranking across environments. Complementary analyses, including AMMI or GGE biplots, may assist in visualising genotype stability, whereas genomic prediction models incorporating G×E effects provide an opportunity to evaluate whether environmental information improves prediction accuracy relative to conventional genomic selection models. The principal outcomes of this framework should include trait-specific SB-to-field genetic correlations, genotype-rank concordance, selection coincidence between SB and field conditions, and comparative prediction accuracy across environments.

Implementation of such a standardised validation framework would establish an empirical bridge between accelerated generation advancement and realised field performance, thereby addressing one of the most important knowledge gaps currently limiting practical deployment of GASB in cotton breeding. Adoption of comparable validation protocols across breeding programmes would also facilitate cross-study comparisons, strengthen the evidence base for SB implementation, and accelerate the integration of genomics-assisted speed breeding into routine cotton improvement. Although this framework is intended as a general reference rather than a prescriptive protocol, it provides a practical guide for designing future validation studies across diverse agroecological conditions.

## Resource-based starter guide for GASB implementation

**Figure 4:**
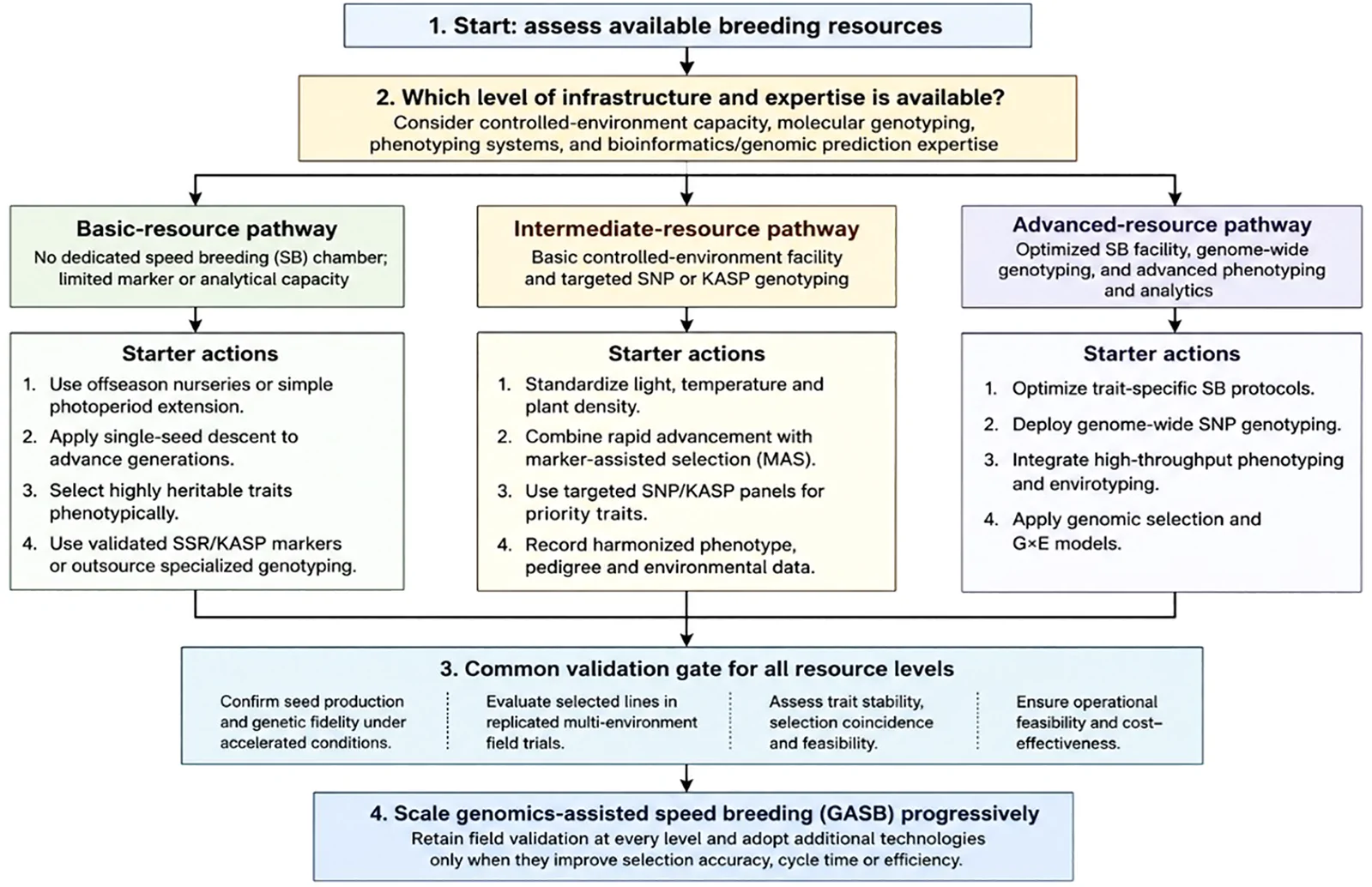
Resource-based starter guide for implementing genomics-assisted speed breeding in cotton. Breeding programmes are grouped into basic-, intermediate-, and advanced-resource pathways according to their controlled-environment, genotyping, phenotyping, and analytical capacities. Each pathway provides a simplified sequence of initial implementation actions, while all pathways converge on a common validation gate requiring confirmation of genetic fidelity and replicated multi-environment field evaluation.

Breeding programmes differ substantially in controlled-environment infrastructure, genotyping capacity, phenotyping systems, bioinformatics expertise, and financial resources. Therefore, genomics-assisted speed breeding should be implemented through a progressive rather than uniform pathway. We propose three practical entry levels—basic, intermediate, and advanced—according to the resources available to each breeding programme (**Figure 4**).

Basic-resource programmes may begin with offseason nurseries or simple photoperiod extension, single-seed descent, phenotypic selection for highly heritable traits, and a limited set of validated SSR or KASP markers, with specialised genotyping outsourced where necessary. Intermediate-resource programmes can combine standardised controlled-environment generation advancement with marker-assisted selection, targeted SNP or KASP panels, and harmonised phenotypic, pedigree, and environmental data collection. Advanced-resource programmes can integrate optimised SB protocols with genome-wide genotyping, high-throughput phenotyping, envirotyping, genomic selection, and genotype × environment prediction models.

Regardless of the initial resource level, all accelerated materials should pass through a common validation gate comprising confirmation of seed production and genetic fidelity, followed by replicated multi-environment field evaluation. Additional technologies should be adopted progressively only when they improve selection accuracy, generation time, or operational efficiency.

**Figure 4** summarises a practical implementation pathway rather than a prescriptive protocol. The framework is intended to assist breeding programmes with different levels of infrastructure in progressively adopting GASB technologies according to available resources while maintaining common validation standards.

## Practical seed-to-seed SB protocols for *Gossypium* spp.

## High-throughput phenotyping in cotton GASB

High-throughput phenotyping (HTP) addresses a constraint that speed breeding alone cannot resolve: accelerating generation cycling does not reduce the time or labour required to measure complex traits that determine selection decisions. In a GASB programme operating at four to five generations per year, phenotyping throughput — rather than generation interval — becomes the primary bottleneck unless measurement is automated, parallelised, and integrated with the breeding pipeline. HTP resolves this constraint by enabling non-destructive, multi-trait measurement of large plant populations at early developmental stages, thereby substituting automated image-derived data for labour-intensive, late-stage field observations.

Within the breeder’s equation (ΔG = i × r × σA/L), HTP contributes through two mechanistically distinct pathways. First, HTP raises selection intensity (i) by enabling phenotypic screening of substantially larger populations per unit time than is feasible with manual methods, increasing the proportion of evaluated individuals from which the superior fraction is selected. Second, HTP improves selection accuracy (r) by providing early-stage proxy measurements that predict mature-plant quantitative traits — fibre length, micronaire, drought tolerance, yield potential — that cannot be directly observed within the compressed SB cycle. These contributions are independent of, and complementary to, the reduction in generation interval (L) achieved through SB and IER, which is why HTP constitutes a mechanistically distinct pillar of the GASB integration framework rather than a component of genomic selection.

Five HTP applications have been experimentally validated in *Gossypium hirsutum* and are directly applicable to GASB programmes. Leaf hairiness — a quantitative trait associated with fibre yield, fibre value, and insect resistance — has been scored at scale using HairNet, a convolutional neural network achieving human-expert-level accuracy on field canopy images, eliminating the requirement for manual trichome counting across large segregating populations (Rolland et al., 2022). AI-assisted image analysis integrating RGB imaging with physiological validation has demonstrated robust progressive drought detection capacity across a diverse *G. hirsutum* germplasm panel, enabling early identification of stress-tolerant individuals before visible symptoms appear (Renó et al., 2024). Cotton leaf chlorophyll content — which directly reports nitrogen status and photosynthetic capacity and is the primary target of the SPAD monitoring protocol described in the Nutritional Physiology section — has been predicted from *in situ* hyperspectral reflectance using machine learning, providing a non-destructive high-frequency alternative to SPAD meter readings (Thorp et al., 2024). Maximum quantum efficiency of photosystem II (Fv/Fm), a sensitive early indicator of photoinhibitory and drought-induced stress at the squaring and boll development stages, has been predicted with high accuracy from hyperspectral imaging data using one-dimensional convolutional neural networks (1D-CNN), enabling population-level stress screening without fluorometry (Wu et al., 2022). Fruit branch angle — a determinant of canopy architecture, boll accessibility, and harvest efficiency — was characterised across 355 upland cotton accessions using a deep-learning-assisted GWAS pipeline that combined aerial image phenotyping with genome-wide association analysis, simultaneously accelerating trait measurement and genetic dissection (Li et al., 2024).

**Figure 5:**
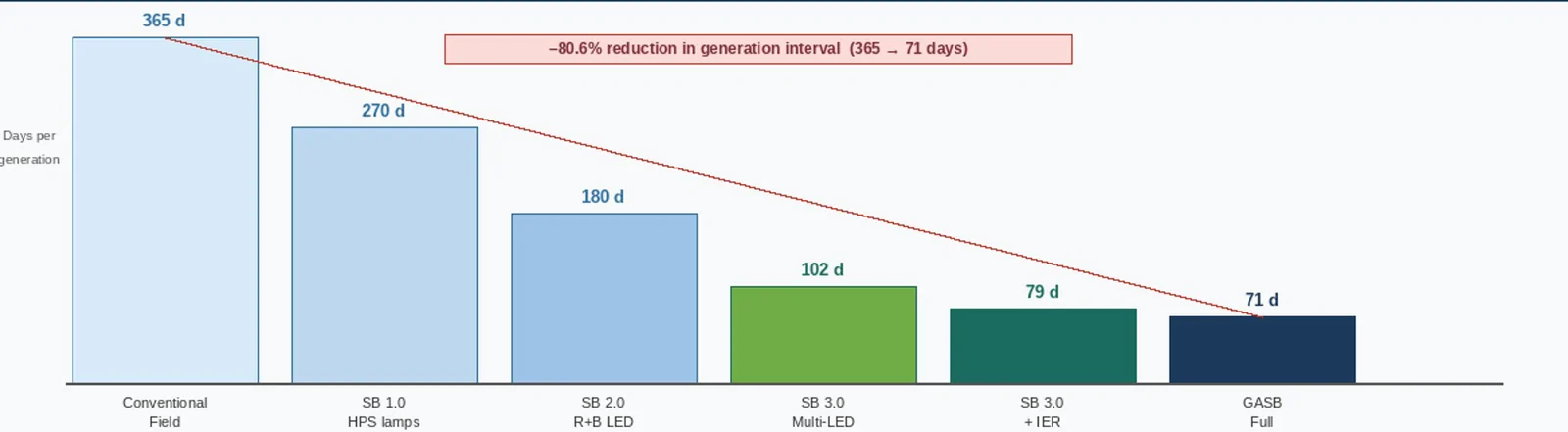
SB technology progression and generation interval compression in Gossypium spp. Each technology addition provides a distinct mechanistic contribution to generation interval reduction. The 80.6% overall reduction — from 365 d (conventional field) to 71 d (GASB with IER) — is achieved cumulatively: photoperiod extension (SB 1.0), multi-channel spectral optimisation (SB 3.0), IER (+IER), and molecular selection integration (GASB). The red trend line illustrates the compression trajectory.

Across these validated applications, a consistent operational logic emerges: traits that require destructive sampling, mature boll development, or specialised laboratory equipment under conventional phenotyping become predictable from non-destructive, early-stage image or spectral measurements under HTP. This substitution is essential for GASB programmes where each accelerated generation represents a significant resource investment and where selection decisions must be made before trait expression is complete (**Figure 5**). Sensor systems currently applicable to cotton SB chambers include RGB cameras for plant architecture and developmental staging, thermal infrared cameras for canopy temperature monitoring — directly supporting the 33°C pollen safety ceiling established in the Thermal Biology section — near-infrared and hyperspectral sensors for pigment, water status, and stress detection, and time-of-flight or structured-light 3D imaging for volumetric growth analysis. Standardised HTP protocols, including calibration procedures, QC thresholds, and data management pipelines, are necessary prerequisites for integration with genomic selection models, as phenotypic data quality directly determines GEBV prediction accuracy across SB generations.

## AI-enabled phenotyping and digital twins for cotton GASB

Artificial intelligence and digital twins represent complementary but distinct components of an advanced GASB platform. AI methods, including computer vision, machine learning, and deep learning, extract phenotypic information and generate predictions from images, sensor data, genomic profiles, and environmental measurements. A digital twin extends this concept by creating a dynamic virtual representation of a physical plant, population, growth chamber, or field environment that is repeatedly updated as new observations become available. In contrast to a static crop simulation model, a functional digital twin links the physical and virtual systems through recurrent data assimilation and, at its most advanced level, uses model outputs to modify management or experimental conditions. Agricultural systems labelled as digital twins nevertheless vary in maturity, and many current applications are more accurately described as digital models or digital shadows because they support monitoring and prediction without fully automated bidirectional control.

Concrete examples from other crops demonstrate how this framework could support cotton GASB. In greenhouse tomato, a virtual production system combined the KASPRO greenhouse-climate model with the INTKAM tomato-growth model. Real greenhouse construction, weather, climate-control settings, crop architecture, and management data were used to simulate photosynthesis, biomass partitioning, fruit development, and fresh yield. The calibrated virtual greenhouse was then used to test alternative lighting duration, temperature, and CO_2_-control scenarios before their implementation in the physical greenhouse. This example is directly relevant to cotton SB chambers, where comparable simulations could evaluate photoperiod, PPFD, spectral composition, temperature, humidity, and plant-density combinations without exposing entire breeding populations to untested regimes.

Maize studies provide a second transferable model. Sequential leaf-area-index measurements derived from UAV imagery have been assimilated into the WOFOST crop model using an Ensemble Kalman Filter, substantially improving in-season growth simulation and yield prediction relative to non-assimilated models. In one two-year study, UAV-derived LAI assimilation reduced yield-prediction RMSE from 413 to 132 kg ha^-^¹ in one season and from 392 to 215 kg ha^-^¹ in the second. More recent work combining machine-learning-derived UAV traits with crop simulation similarly illustrates how continuously updated phenotypic observations can constrain uncertain model states. These systems are not always full bidirectional digital twins, but they provide the essential digital-shadow architecture on which a breeding-oriented twin can be developed.

A related maize–soybean framework integrated UAS multispectral imagery, deep-learning estimates of above-ground biomass or LAI, and DSSAT crop models through data assimilation. The resulting models achieved grain-yield prediction accuracies of R² = 0.62 for maize and R² = 0.81 for soybean, demonstrating the value of combining sensor-derived phenotypes, AI, and process-based crop simulation. Such an architecture could be adapted to cotton by integrating RGB, thermal, hyperspectral, and 3D imaging with chamber environmental data and genomic information to update predictions of flowering time, plant architecture, stress response, boll retention, and subsequent field performance.

For cotton GASB, a practical digital-twin architecture could be developed in four stages. First, chamber sensors and imaging systems would continuously capture temperature, humidity, PPFD, spectral composition, canopy temperature, plant height, leaf area, developmental stage, and reproductive retention. Second, AI models would convert raw images and sensor streams into standardised phenotypic traits. Third, these observations would be assimilated into crop-growth, genomic-prediction, or hybrid physiological–statistical models to update genotype-specific growth trajectories and estimate the probability of successful flowering, seed set, stress tolerance, and field performance. Fourth, the virtual model would compare alternative scenarios and recommend changes in photoperiod, temperature, irrigation, nutrition, plant density, or harvest timing. Only after prospective validation should these recommendations be linked to automated chamber control.

The immediate value of this approach is not the creation of a fully autonomous virtual cotton plant, but the progressive development of a validated digital shadow that improves experimental repeatability and decision quality. A realistic first target would be an AI-assisted chamber twin that predicts flowering and boll maturity from time-series images and environmental data. Subsequent versions could integrate genomic estimated breeding values, multi-environment field observations, and G×E models to rank lines and test virtual management scenarios. Major limitations include the need for large, standardised longitudinal datasets, genotype-specific calibration, interoperability between sensor and breeding databases, uncertainty quantification, and independent SB-to-field validation. Therefore, AI and digital-twin outputs should initially support, rather than replace, breeder decisions.

## Critical analysis: challenges, limitations, and unresolved questions

## Economic considerations for implementing cotton GASB

Although the initial investment required for genomics-assisted speed breeding (GASB) may appear substantial, its economic feasibility should be evaluated over the entire breeding pipeline rather than individual technology components. The principal economic advantage of GASB arises from shortening the breeding cycle, thereby reducing the time required to release improved cultivars while increasing annual genetic gain. Investments in controlled-environment facilities, molecular genotyping, high-throughput phenotyping, and data management are partially offset by fewer field generations, reduced land and labour requirements per breeding cycle, earlier elimination of inferior lines, and faster delivery of elite cultivars to growers.

The economic return is expected to vary according to breeding-programme scale and available infrastructure. Basic-resource programmes may achieve meaningful reductions in generation interval using simple photoperiod extension and limited marker-assisted selection with relatively modest capital investment. Intermediate programmes benefit from standardised controlled-environment facilities and targeted SNP or KASP genotyping, whereas advanced programmes can further improve selection efficiency through genome-wide genotyping, genomic prediction, AI-assisted phenotyping, and integrated data management. Consequently, the incremental adoption of GASB technologies provides a practical strategy for maximizing return on investment while minimising unnecessary capital expenditure.

Rather than recommending a universal implementation model, breeding programmes should evaluate each technology according to its contribution to generation-time reduction, selection accuracy, operational efficiency, and long-term genetic gain. Progressive adoption based on demonstrated biological and economic benefits is therefore likely to provide the most sustainable pathway for routine GASB implementation. Because capital, energy, labour, land and genotyping costs vary substantially among countries and institutions, programme-specific feasibility assessments should include local cost accounting and sensitivity analysis of the principal economic drivers before major infrastructure investments are made.

## Future perspectives: GASB roadmap 2025–2035

The 2025–2035 decade will determine whether GASB becomes the operational standard for cotton variety development or remains a demonstration technology. The critical inflection point is 2027: programmes that have commissioned bioinformatics infrastructure, validated their KASP Level 2 panel, and initiated SB-to-field correlation studies by that point will be positioned to nominate their first GASB-selected varieties for SVT by 2030. The consequence for those that do not invest during 2025–2027 is a widening productivity gap relative to GASB-enabled programmes in China, Australia, and the United States.

The 2025–2027 near-term priorities in **Table 12** are not aspirational — they are executable with existing capacity in well-equipped national cotton research programmes. The multi-genotype G×L study, KASP Level 2 panel deployment, and SB-to-field correlation study are the three critical-path items that determine whether the medium-term GASB pipeline can be launched on schedule.

**Table 12:** Prioritised GASB development roadmap for cotton breeding, 2025–2035.

Horizon	Domain	Priority action	Key performance indicator
2025–2027 Near Term	Protocols	Multi-genotype G×L study: ≥20 accessions × 4 LED recipes × statistical validation	Validated SB protocol published for ≥10 representative *Gossypium* genotypes (≥5 elite cultivars and ≥5 landrace accessions), with genotype × light interaction quantified by 2027.
	Markers	KASP Level 2: deploy heat (D06-5486215) + drought (22079/22089) + salt + early maturity panel	Multi-trait KASP panel validated for heat, drought, salinity and earliness; routine screening capacity ≥500 genotypes week^-^¹ at <$0.50 per sample
	Infrastructure	GBS-capable bioinformatics pipeline (HPC + GPU + NAS or cloud)	Operational genomic-selection pipeline established and prospectively validated on ≥200 elite breeding lines by 2027
	Validation	Complete replicated validation across ≥4 environments and publish SB–field correlation coefficients for ≥6 agronomic traits by 2027.	Published SB–field correlation coefficients (r and r²) for ≥6 agronomic traits
	Protocol	LED-only 4-gen/yr standard operating procedure (no IER), fully statistically validated	Adopted by ≥3 breeding programmes without tissue culture infrastructure
2027–2030 Medium Term	GASB pipeline	Full KASP MAS + GEBV integration each SB generation, incorporating G×E GS models	At least one breeding population advanced to official multi-location testing using the GASB pipeline
	G. *barbadense*	SB protocol for SD-sensitive *G. barbadense* via staged photoperiod + Gb_Ppd1 KASP	Validated staged-photoperiod protocol achieving ≥3 generations year^-^¹ across ≥3 G. *barbadense* breeding populations
	HTP	Deep-learning fibre quality prediction from seedling or early-boll imaging	Prediction accuracy ≥0.70 for fibre length and ≥0.65 for micronaire using early-generation imaging
	Policy	Evidence-based SVT reform: streamlined pathway for GASB-derived varieties	National recommendations for evaluation of GASB-derived cultivars submitted to the national variety testing authority
	Capacity	≥10 breeders trained in GASB at ≥2 national institutes	≥20 breeders trained and ≥3 national breeding institutions implementing standardised GASB protocols
2030–2035 Long Term	NGT editing	Transgene-free base/prime editing of GhLUX1/GhELF3/GhCOL3 in elite backgrounds	Transgene-free edited lines confirmed by sequencing and advanced to BC_2_ or later generations
	AI-assisted phenotyping	Develop a validated phenotyping pipeline processing ≥1000 plants day−1 with standardised QC by 2030.	Validated AI phenotyping pipeline processing ≥1000 plants day^-^¹ and prospectively validated across ≥3 breeding cycles by 2035
	Regional network	Regional GASB network: Uzbekistan, Kazakhstan, Turkmenistan, Pakistan, India	Regional GASB network established with ≥4 participating institutions, harmonised phenotyping protocols and annual multi-location germplasm exchange

All milestones are intended as indicative targets for guiding future GASB development rather than mandatory performance thresholds and should be adapted according to programme objectives and available resources.

## Technology accessibility and equitable implementation

Although genomics-assisted speed breeding (GASB) offers substantial opportunities to accelerate cotton improvement, its successful implementation will depend not only on scientific advances but also on equitable access to enabling technologies. Many breeding programmes in developing regions face limitations in controlled-environment facilities, molecular genotyping, bioinformatics infrastructure, and skilled personnel. Therefore, widespread adoption of GASB should emphasise scalable implementation strategies, regional collaboration, shared research infrastructure, open-access analytical tools, and capacity-building initiatives.

The resource-based implementation framework proposed in this review is intended to facilitate progressive adoption according to local infrastructure and financial capacity rather than requiring simultaneous deployment of all advanced technologies. International partnerships should also address responsible germplasm exchange, data ownership, intellectual-property arrangements, and fair sharing of benefits arising from locally adapted genetic resources. Improving technology accessibility through collaborative networks and knowledge sharing will be essential to ensure that the benefits of accelerated breeding are distributed across both well-resourced and resource-limited breeding programmes.

Promoting equitable access to breeding technologies will contribute not only to scientific progress but also to climate resilience, sustainable agricultural development, and the long-term capacity of developing regions to maintain locally responsive cotton-breeding programmes.

## Conclusions

### Speed breeding is not a standalone technology

It is the temporal engine of an integrated next-generation cotton breeding system, and its value is proportional to the quality of the molecular selection decisions made within each compressed generation. A programme implementing SB without simultaneously deploying KASP foreground selection and building toward genomic selection will accelerate cycling without accelerating genetic gain. This reflects the fact that for mature-stage yield, fibre quality, and certain adult-plant resistance traits, the capacity for direct phenotypic selection at the seedling stage is inherently limited. In contrast, several early-development traits—including seedling vigour, root architecture, chlorophyll-related characteristics, canopy or leaf temperature, and early stress responses—remain amenable to phenotypic evaluation and can provide valuable complementary information for breeding. Consequently, molecular selection remains essential for efficiently advancing traits that cannot be directly evaluated during the seedling stage, thereby reducing reliance on mature-stage phenotyping during rapid generation advancement and helping to alleviate the phenotyping bottleneck that speed breeding is intended to address. The six GASB pillars reviewed here are each independently beneficial and multiplicatively powerful in combination. The molecular architecture of flowering control (Section 2) identifies the *GhFKF1*, *GhCOL3*, *GhLUX1*, and *GhELF3* targets whose manipulation through LED recipe design or NGT editing directly controls generation interval at the molecular level. LED spectral optimisation (Section 3) converts this molecular knowledge into engineered photon environments through the mechanistically non-redundant four-channel architecture, while thermal management (Section 4) protects reproductive output — the 33°C pollen safety ceiling is the most operationally critical single parameter in any cotton SB facility. Nutritional physiology and biostimulants (Section 5) deliver additional generation-interval compression through metabolic acceleration. *In vitro* biotechnology (Section 6) provides the IER pathway to five-generation-per-year cycling and the NGT transformation gateway, Operational coordination between pollen-stage temperature management and immature embryo rescue is required when different breeding populations are maintained simultaneously within speed-breeding facilities. Molecular genomics (Section 7) provides selection precision — from KASP foreground MAS through GS models with 0.65–0.76 prediction accuracy for fibre traits — that converts each compressed generation into a high-accuracy selection decision, while the SB-to-field correlation gap identified in Section 9 remains the most urgent unresolved scientific question for GASB field deployment.

**Figure 6:**
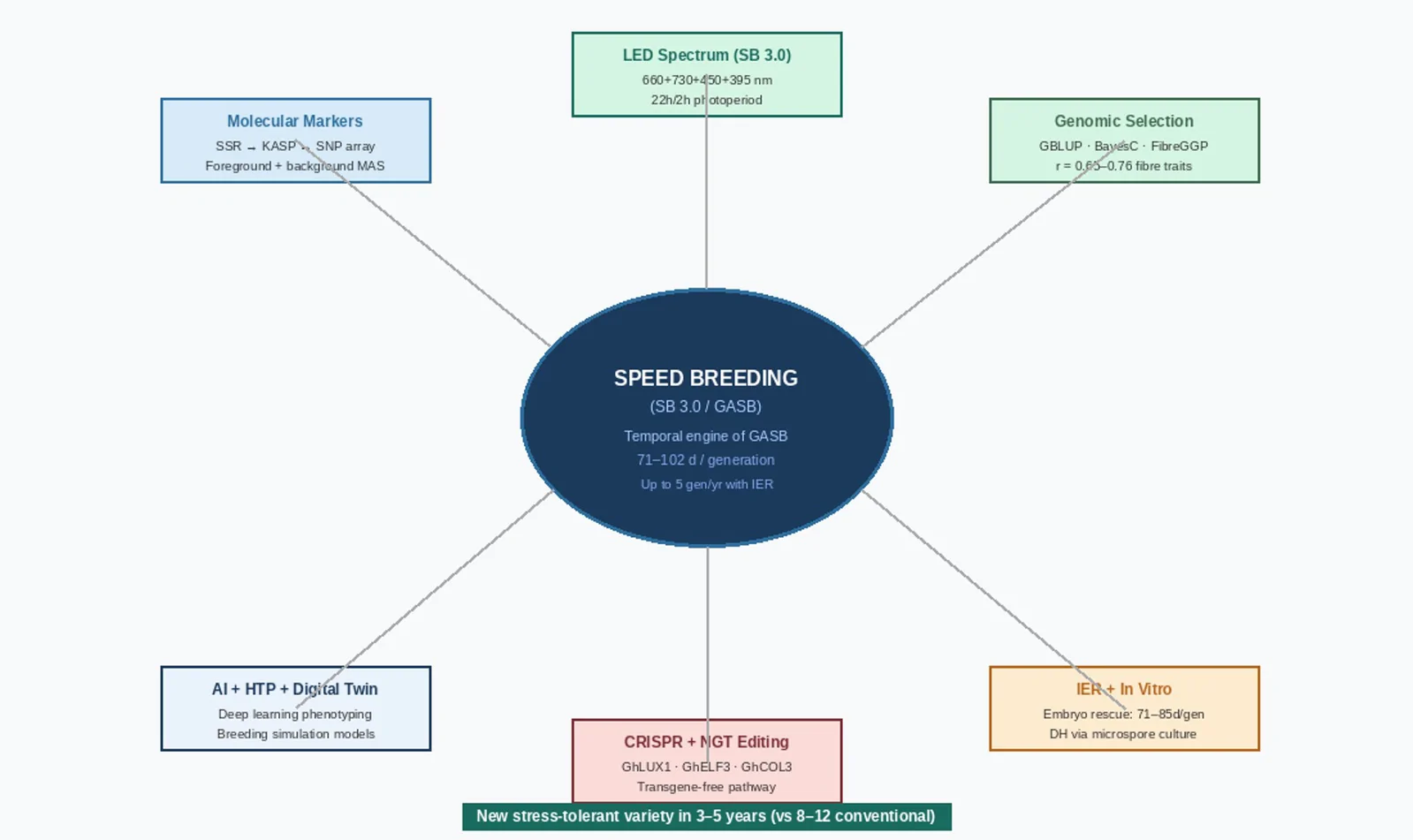
The GASB Integration Hub: speed breeding as the central platform connecting six technology pillars. Each spoke represents a mechanistically distinct contribution to the four components of the breeder’s equation (ΔG = (i × r × σA)/L). Integration of all six pillars achieves multiplicative improvement in annual genetic gain. The target output — a new stress-tolerant, high-fibre-quality cotton variety in 3–5 years vs. 8–12 years conventional — is achievable with currently demonstrated technology components. NGT, New Genomic Technique; IER, Immature Embryo Rescue; HTP, High-Throughput Phenotyping (five cotton-validated applications reviewed in the dedicated HTP section); AI, Artificial Intelligence (digital twin architecture and cotton GASB decision-support framework reviewed in the dedicated AI section).

High-throughput phenotyping and AI-assisted decision tools — reviewed with five cotton-experimentally validated HTP applications and a practical digital twin architecture in dedicated sections — contribute to the breeder’s equation through two pathways that are mechanistically independent of generation interval compression: HTP raises selection intensity (i) by enabling phenotypic screening of substantially larger populations per unit time, and AI-assisted prediction raises selection accuracy (r) by substituting model-based GEBV estimates and image-derived proxies for late-stage field phenotyping that is incompatible with the compressed SB timeline. These contributions complete the mechanistic rationale for HTP and AI as independent pillars of the GASB Integration Hub (**Figure 6**), rather than supplementary technologies appended to the genomic selection framework.

Six operationally specific conclusions: (1) The four-channel LED recipe is mechanistically non-redundant: each channel targets a distinct photoreceptor interface and cannot be omitted without specific performance loss. (2) Canopy IR thermometry is mandatory: the 33°C pollen ceiling operates without visual warning signs. (3) Operational scheduling is required to coordinate pollen-stage temperature management and immature embryo rescue when different breeding populations are maintained simultaneously within controlled-environment facilities. (4) NGT editing and SB are temporally sequential, not simultaneous: edited events must complete transformation before entering the SB-MABC pipeline. (5) GASB must begin at Level 1 immediately and scale to Level 3 incrementally — waiting for complete infrastructure forfeits years of achievable genetic gain. (6) SB-to-field correlation must be measured, not assumed — systematic multi-environment validation studies, incorporating genomic G×E prediction models, are the foundation on which the regulatory case for streamlined SVT must be built.

*Gossypium* spp. faces compounding stresses from heat, salinity, and water scarcity on timescales that make the 8–12 year conventional pipeline a strategic liability. The scientific foundations of a 3–5 year GASB pipeline are established, as demonstrated by the 1.5-year BC4F3 result of Wang et al. (2025). The biological barriers are known and mitigable. The institutional barriers — bioinformatics infrastructure, KASP panel commissioning, regulatory reform, and human capital — are addressable within planned investment horizons. The question is not whether cotton breeding can be transformed, but how rapidly the institutional commitment to implement that transformation will be realised.

Future studies should determine whether the protocols developed for *G. hirsutum* can be adapted and experimentally validated for *G. barbadense*, semi-wild accessions, landraces, and other *Gossypium* species under comparable controlled-environment conditions. 
