# Supplementary material for: Speed breeding in upland cotton (Gossypium hirsutum L): scientific foundations and integrated implementation of a next-generation cotton breeding platform
Source: Front Plant Sci. 2026 Sep 7;17:1915693. doi: 10.3389/fpls.2026.1915693 (PMC13591094; doi:10.3389/fpls.2026.1915693)
Supplement: Supplementary file 1 [file Table1.docx]

***Figure S1. Detailed implementation workflow for genomics-assisted speed breeding (GASB) in cotton. The diagram illustrates stepwise implementation options according to available breeding resources and infrastructure, from initial adoption to routine deployment following multi-environment validation.***
